# Effects of Freeway Rerouting and Boulevard Replacement on Air Pollution Exposure and Neighborhood Attributes

**DOI:** 10.3390/ijerph16214072

**Published:** 2019-10-23

**Authors:** Regan F. Patterson, Robert A. Harley

**Affiliations:** 1 Department of Civil and Environmental Engineering, 607 Davis Hall, University of California, Berkeley, CA 94720, USA; 2 Department of Civil and Environmental Engineering, University of California, Berkeley, CA 94720-1710, USA; harley@ce.berkeley.edu

**Keywords:** freeways, boulevards, urban green space, transportation, traffic-related air pollution, environmental gentrification

## Abstract

Freeway rerouting and replacement with a street-level boulevard are urban transportation policies, that may help redress disproportionate air pollution burdens resulting from freeway construction that took place during the mid-20th century. However, environmental justice activism for freeway rerouting and urban green space creation may have the unintended consequence of environmental gentrification. In this paper, we investigate the effects of freeway routing decisions on exposure to traffic-related air pollution and neighborhood socioeconomic and demographic change. We focus on the effects of rerouting the Cypress Freeway in West Oakland, along with the construction of a street-level boulevard (Mandela Parkway), on the original freeway alignment. The impacts of two rebuild scenarios, freeway rebuild-in-place and reroute, on near-roadway NO_x_ and BC concentrations are compared. We also assess changes in demographics and land use in West Oakland, between the time when the Cypress Freeway was damaged by a major earthquake and after completion of Mandela Parkway. Our research indicates that freeway rerouting reduced annual average concentrations of both NO_x_ (−38% ± 4%) and BC (−25% ± 2%) along the Mandela Parkway alignment. However, there is evidence of environmentally driven neighborhood change, given that there are larger decreases in the long-time Black population (−28%) and increases in property values (184%) along Mandela Parkway, compared to West Oakland as a whole. There are some attributes along the Mandela Parkway that enable low-income residents to live in proximity to the street-level boulevard, such as affordable housing.

## 1. Introduction

The Federal-Aid Highway Act of 1956 called for the construction of 41,000 miles of interstate highway by 1970 and created the Highway Trust Fund to finance it. From the mid-1950s to the early 1970s, the Interstate Highway System transformed US urban landscapes. Urban planners saw the urban freeway as a solution to growing traffic congestion in cities, as well as a tool to achieve the urban renewal goal of “slum” clearance [1,2]. Planners and engineers decided where freeways would be built, with little to no citizen oversight [3]. Freeway construction resulted in the demolition, division, and forced removal of poor communities of color, particularly African-Americans [1,4,5]. Freeways facilitated white flight and accelerated white suburbanization [6,7], reinforced racial residential segregation [6,8], and increased air and noise pollution [9,10], mostly in communities of color. Racial borders achieved through discriminatory race-based planning processes, such as redlining, restrictive covenants, and zoning [11,12,13,14,15], were concretized into the built environment with freeway construction. The adverse effects of freeway construction are environmental justice issues [9].

Freeway removal or rerouting is viewed as an opportunity to redress the health and environmental impacts of freeway construction [2,16]. Teardown advocates seek to reroute freeways through alternative corridors or bury them in tunnels or trenches. For communities near freeways, this practice has significant implications for addressing traffic-related air pollution and associated adverse health effects, including asthma exacerbation, lung impairment, impacts on fertility and birth outcomes, and cardiovascular and respiratory mortality [17,18,19]. A quantitative analysis of the air quality benefits of freeway removal is needed. Projects often involve building street-level boulevards in the former corridor. Transforming the former freeway alignment into a landscaped boulevard also increases urban green space. Green space has health benefits, such as increased physical activity and psychological well-being [20]. However, a potential unintended consequence of efforts to expand urban green space is the green space paradox [20]. Urban green space, aimed at addressing environmental injustice, can make a neighborhood more desirable, potentially leading to gentrification and the displacement of the residents for whom the green space was created. This paradoxical situation has been termed environmental gentrification [21]. Studies indicate that freeway removal or tunneling can increase property values [2,22,23,24]. However, the relationship between freeway removal and gentrification is largely unexplored in the peer-reviewed literature. One study assessed the change in neighborhood racial composition along an old freeway alignment replaced with a boulevard and found an increase in the percentage of white population and a decrease in the percentage of Black population [23]. More research is needed to investigate how freeway removal or rerouting, and conversion of the old alignment to a boulevard, affect demographic and socioeconomic change.

In West Oakland, residents successfully advocated for rerouting the Cypress Freeway and creating a street-level boulevard along the original alignment. West Oakland, a redlined neighborhood [25] and one of the few East Bay neighborhoods where African-Americans could own homes [26], was targeted for, and adversely affected by, freeway construction. In 1958, the elevated, double-decked Cypress Freeway (I-880) was completed. It bisected West Oakland and physically segregated the neighborhood. Construction of the Cypress Freeway led to property demolitions and displaced 600 families [27]. The later-constructed Grove Shafter (I-980) and MacArthur (I-580) Freeways further segregated the neighborhood. Freeway construction and other urban renewal projects in West Oakland destroyed over 5000 housing units and resulted in economic decline in the area [26].

**Figure 1:**
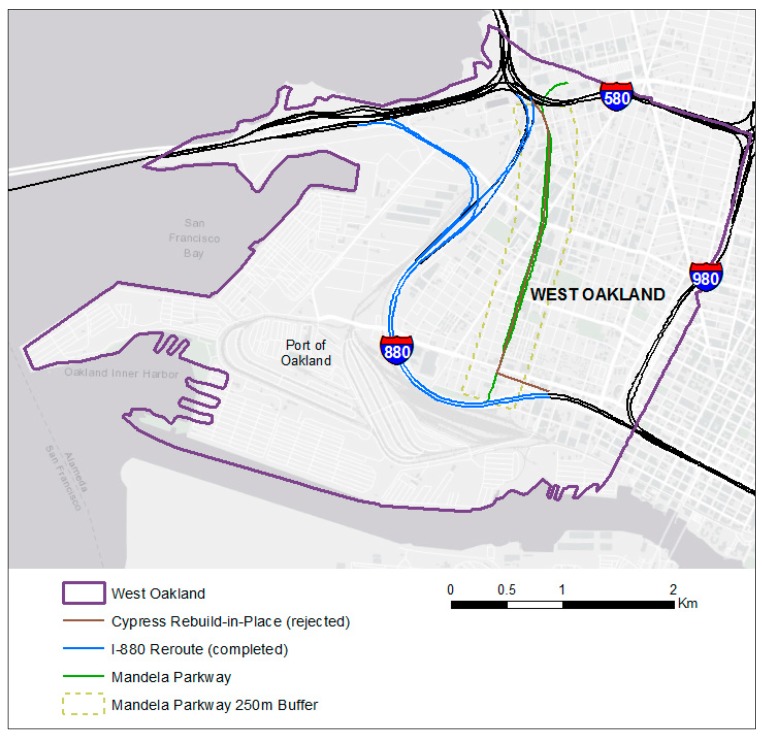
Map of West Oakland study area.

When the Cypress Freeway collapsed during the 1989 Loma Prieta earthquake, the California Department of Transportation (Caltrans) favored a rebuild option on the same alignment [16]. However, legislation such as the National Environmental Protection Act of 1969 provided the community with the opportunity to participate in the decision-making process, an option that was not previously available. Community activists organized to oppose reconstruction along the original route and redress economic and environmental justice issues [16]. After the public comment period for the draft environmental impact statement closed in 1991, Caltrans selected an alternative route around the perimeter of West Oakland, in an industrial area (Figure 1). Some felt the proposal did not adequately address local concerns and filed a discrimination suit, under Title VI of the Civil Rights Act of 1964 [16]. The case, *Clean Air Alternative Coalition v United States Department of Transportation*, was settled out of court and resulted in several additional mitigation measures [16,28], including the transformation of the former Cypress Freeway route into a landscaped boulevard, later named Mandela Parkway. Construction of Mandela Parkway began in 2002 and was completed in 2005.

In this study, we investigate the air pollution and neighborhood impacts of rerouting the Cypress Freeway and constructing a street-level boulevard in West Oakland. Our specific objectives are to: (i) quantify the local effects on air pollution of rerouting the Cypress Freeway, through modeling near-roadway concentrations for two different rebuild scenarios, and (ii) examine neighborhood socioeconomic and demographic impacts, as reflected by spatiotemporal changes in indicators of gentrification, to assess whether existing residents benefit from the freeway-to-boulevard conversion, or are excluded through the phenomenon of environmental gentrification.

## 2. Methods

### 2.1. Exposure to Traffic-Related Air Pollution

The Cypress Freeway collapsed in 1989, and construction of the rerouted freeway and Mandela Parkway were completed in 1998 and 2005, respectively. We assessed two freeway routing scenarios: (1) rebuild-in-place—the rejected plan to reconstruct the damaged Cypress Freeway on the original alignment, which divided a residential neighborhood in West Oakland—and (2) reroute—the completed plan, which involved reconstructing the freeway along a different route (I-880 reroute) to circle around, rather than bisect, a residential neighborhood in West Oakland, and replacing the removed section of the freeway with a street-level boulevard (Mandela Parkway). Near-roadway concentrations of nitrogen oxides (NO_x_) and black carbon (BC) are estimated along the Cypress Freeway rebuild-in-place and Mandela Parkway for the year 2009. This year was selected due to the availability of traffic count data for Mandela Parkway. West Oakland is a port-adjacent community (Figure 1) that is heavily impacted by heavy-duty truck traffic [29]. Heavy-duty trucks are major sources of NO_x_ and BC [30,31,32].

#### 2.1.1. Traffic Volumes

Road network data for the I-880 reroute and Mandela Parkway were downloaded as shapefiles from the California Department of Transportation [33] and the City of Oakland [34], respectively. Traffic count data for Mandela Parkway are from the West Oakland Truck Survey [35], which provides manual truck survey counts and automatic vehicle counter data at three locations along Mandela Parkway, made in August, 2008. The automatic counters characterized the vehicle fleet mix, including proportions of light-duty vehicles, buses, and trucks by number of axles. This enabled us to estimate counts for other vehicle types from the manual truck counts. To align with EMFAC [36] vehicle types, two-axle/six-tire truck counts were mapped to light-heavy and medium-heavy duty trucks, as described elsewhere [37]. Trucks with three or more axles were classified as heavy-heavy duty trucks and all were assumed to be diesel-fueled, and referred to as heavy-duty diesel trucks hereafter [37]. Automatic counter-derived hourly temporal profiles for each vehicle type were used to estimate hourly traffic volumes.

For the Cypress rebuild-in-place scenario, we combined measured traffic volumes for Mandela Parkway and the I-880 reroute, and we assigned that traffic to the original Cypress Freeway route. Traffic data on the I-880 reroute were obtained from the California Department of Transportation. Available data include link-specific counts for total vehicle annual average daily traffic (AADT), proportion of trucks, and truck counts broken down by number of axles. We estimated light-duty vehicle counts by subtracting trucks from total vehicle counts. Two-axle/six-tire trucks and trucks with three or more axles were apportioned as described above. Traffic volumes were mapped to hourly estimates using month-of-year, day-of-week, and hour-of-day temporal allocation factors from McDonald et al. [37].

#### 2.1.2. Vehicle Emissions

Estimates of link-specific emission rates were calculated from hourly traffic volumes and emission factors. NO_x_ and PM_2.5_ emission factors by vehicle type were estimated using EMFAC model outputs at the county level. For Mandela Parkway, emission factors were defined using estimates for running exhaust emissions, with an average speed of 30 mph, which was the average vehicle speed indicated by the automatic traffic counter [35]. For the freeway, emission factors were calculated for aggregated vehicle speeds. We estimated BC emission factors using the EMFAC-derived PM_2.5_ emission factors, combined with gasoline and diesel BC fractions of 18% and 72%, respectively [38,39,40].

#### 2.1.3. Near-Roadway Air Pollutant Concentrations

We predicted traffic-related air pollutant concentrations using the RLINE line-source dispersion model [41,42]. We previously evaluated RLINE model performance in predicting NO_x_ and BC concentrations at near-roadway monitoring sites in the San Francisco Bay Area [43]. The study domain was overlaid with a 50 m grid, and model receptors were set at grid centroids. We modeled concentrations within 250 m of Mandela Parkway and the I-880 reroute, since traffic-related air pollution levels are known to be elevated at distances of up to about 200 m from major roadways [18,44,45]. The meteorological inputs required for RLINE dispersion calculations were developed using AERMET [46], using meteorological data from the National Weather Service for the nearby Oakland International Airport. We ran RLINE using a unit emission rate (1 g m^−1^ s^−1^) at release heights of 0.3 m for light-duty vehicles, and 4 m for heavy-duty trucks [47]. Dispersion model results were combined with hourly emissions estimates to compute emission-weighted NO_x_ and BC concentrations.

Total near-road pollutant concentrations were calculated as the sum of modeled traffic-related and urban background concentrations. Ambient observations at the Bay Area Air Quality Management District (BAAQMD) monitor at West Oakland were used to estimate NO_x_ and BC background levels for this study. To reduce the influence of local NO_x_ emission sources at the background site [48], and characterize the urban background contribution accurately, we defined background NO_x_ concentrations using the 25th percentile method of the West Oakland monitoring site data [49]. Measured BC concentrations were considered representative of urban background concentrations for this pollutant and were used without adjustment. Background concentrations were added to dispersion model estimates of traffic-related air pollution for each modeled receptor within the study domain. Predicted hourly concentrations were then used to compute annual average concentrations at each receptor.

### 2.2. Neighborhood-Scale Changes in Demographics and Land Use

#### Census Data

Data from the 1990 Census, 2010 Census, and the 2006–2010 American Community Survey [50], all calculated to the 2010 tract shapes, were used to investigate the impacts of the freeway rerouting and conversion of the old alignment to a street-level boulevard on neighborhood demographic, socioeconomic, and housing characteristics. Demographic variables include the total population and the percentage of Black, Latino, and nonwhite (i.e., total non-white) residents. Socioeconomic indicators include median household income, percentage of residents with at least a bachelor’s degree, and percentage of residents living in poverty. Housing characteristics include median rent, median home value, and percentage of renter-occupied dwellings.

We compared 1990 and 2010 census variables within 250 m of the Mandela Parkway alignment to corresponding values for all of West Oakland (Figure 1). We used an area-weighting method to estimate the demographic, socioeconomic, and housing composition within a 250 m band for each year. A 250 m buffer was intersected with census tract areas using a Geographic Information System (GIS). The percentage of each census tract’s area within the buffer was computed, and raw census data were weighted using these percentages.

## 3. Results and Discussion

### 3.1. Spatial Distribution of NO_x_ and BC Concentrations

**Figure 2:**
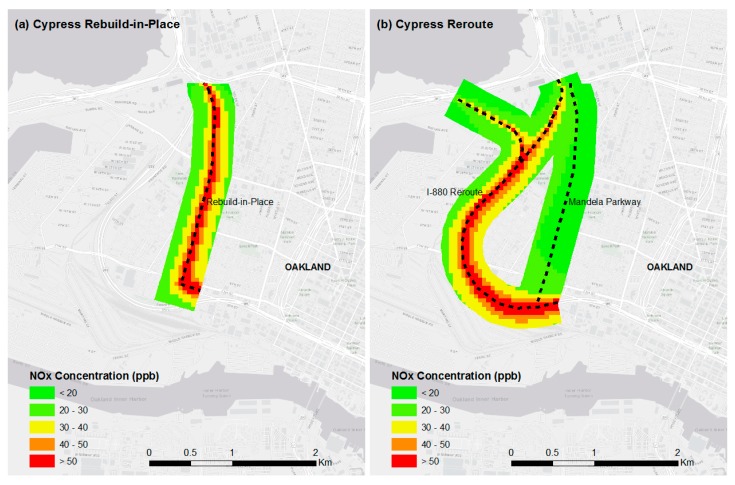

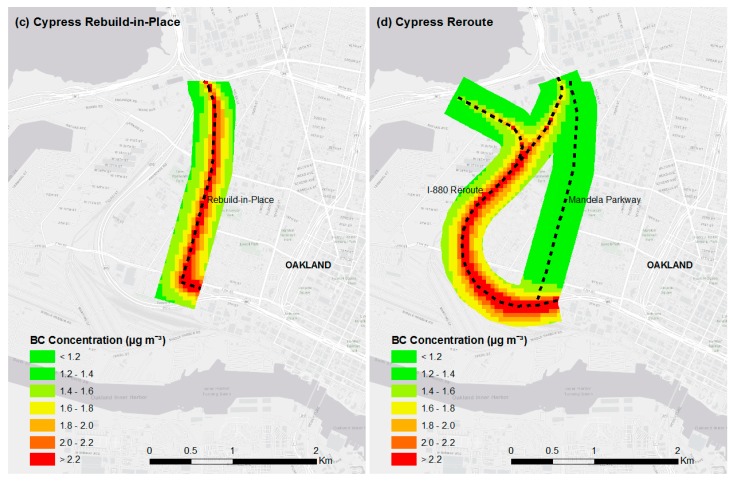
Annual average concentrations in West Oakland for (**a**,**c**) the Cypress rebuild-in-place scenario, and (**b**,**d**) the Cypress reroute scenario. Dashed black lines represent the centerline of each route.

Figure 2 illustrates the impact of the Cypress Freeway rebuild-in-place and reroute scenarios on pollutant concentrations in the middle of West Oakland. The maps in Figure 2 substantiate that by rerouting the Cypress Freeway, high concentrations shift from the middle of West Oakland (Figure 2a,c) to around the periphery (Figure 2b,d). Large pollutant reductions are observed in the middle of West Oakland. For the rebuild-in-place scenario (Figure 2a,c), mean annual average NO_x_ and BC concentrations are 36.1 ± 1.2 ppb and 1.72 ± 0.30 μg m^−3^, respectively. For the reroute scenario (Figure 2b,d), mean annual average concentrations are 22.3 ± 0.8 ppb for NO_x_ and 1.2 ± 0.03 μg m^−3^ for BC.

The estimated pollution reductions highlight the significance of the roadway type routed through residential areas. Concentrations are higher -on the Cypress Freeway rebuild-in-place compared to the Mandela Parkway, as indicated by the visibility of the freeway alignment in Figure 2a,c. Traffic volumes on the Mandela Parkway are substantially lower than those on the Cypress Freeway rebuild-in-place. Traffic volumes on the Cypress Freeway rebuild-in-place range from approximately 108,000 to 126,000 vehicles per day, with heavy-duty diesel trucks accounting for approximately 7% of total traffic. In contrast, Mandela Parkway has average traffic volumes of approximately 500 to 3300 vehicles per day, with heavy-duty diesel trucks accounting for approximately 5% of total traffic. These results provide quantitative support for the expectation that reducing the traffic-carrying capacity through residential areas provides air quality benefits. Replacing a freeway with a boulevard is one transportation policy that reduces the traffic burden in residential areas, particularly regarding heavy-duty diesel trucks. Another such policy is to revise designated truck routes [51].

On average, annual average reductions are larger for NO_x_ (−38% ± 4%) than for BC (−25% ± 2%) in the middle of West Oakland. This may be due to the influence of the I-880 reroute on air pollution near Mandela Parkway, particularly the intersecting segment at the south end, which is evident when comparing the spatial pattern in annual average concentrations, shown in Figure 2b,d versus Figure 2a,c. A stronger influence on BC than NO_x_ is visible in Figure 2b,d, and suggests that BC decays less rapidly than NO_x_, which is consistent with previous studies [52]. These results reveal that, ideally, alternative freeway corridors would not have segments in close proximity to the impacted area of concern. The options for an alternative route were limited in West Oakland, due to existing freeways, ports, and railroads.

### 3.2. Distance–Decay Curves

**Figure 3:**
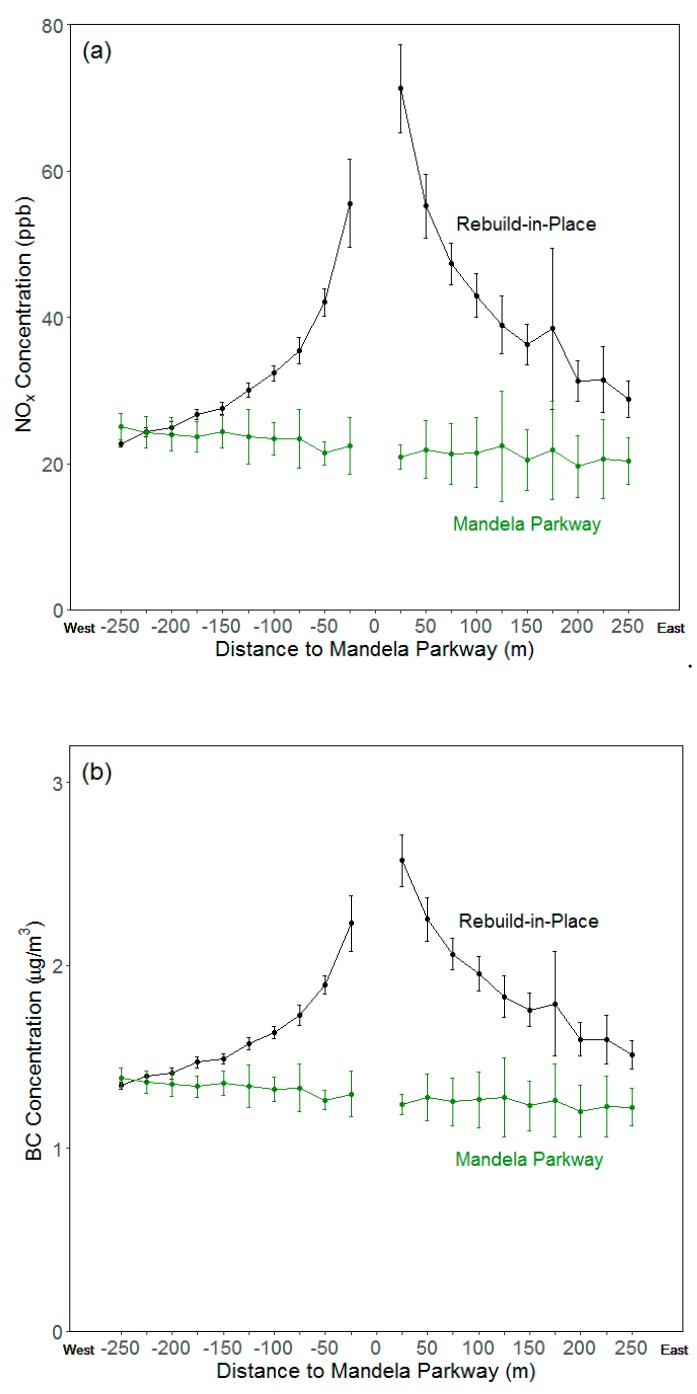
Mean annual average concentrations of (**a**) NO_x_ and (**b**) BC versus distance from the Mandela Parkway alignment. Each data point in the figure represents an averaged value for all measurements within a 25 m distance band. Negative distances are to the west; positive values are to the east. Uncertainty estimates indicate 95% confidence levels.

Figure 3 shows average annual concentrations of NO_x_ and BC at increasing distances from Mandela Parkway. Each data point in the figure represents an averaged value for all model receptors located within 25 m distance bands. For the Cypress rebuild-in-place scenario, we observe much higher pollutant concentrations towards the east at all distances. Mean annual average concentrations of NO_x_ and BC for a distance range of 0 to 25 m east of the freeway are 71.2 ± 6.0 ppb and 2.6 ± 0.1 μg m^−3^, respectively. In contrast, mean annual average concentrations for distances of 0 to 25 m west of the freeway are 55.6 ± 6.0 ppb for NO_x,_ and 2.2 ± 0.2 μg m^−3^ for BC. The concentrations decrease noticeably when moving away from the freeway in both directions, following distance–decay relationships similar to those observed in other studies [45,52]. Pollutants decay at a similar rate on both sides of the Cypress Freeway, with mean NO_x_ concentrations decreasing by 49% ± 9% in the first 150 m east of the freeway, and 50% ± 11% in the first 150 m to the west. BC concentrations decrease by 32% ± 6% and 33% ± 7% in the eastward and westward directions, respectively. The more rapid decline in NO_x_ concentrations relative to the roadway edge is consistent with results from previous studies [52].

Figure 3 further demonstrates the large concentration reductions, observed in Figure 2, that result from rerouting the Cypress Freeway. Annual average pollutant concentrations within 25 m of Mandela Parkway decrease by 66% ± 8% for NO_x,_ and 48% ± 5% for BC, as a result of rerouting. Figure 3 shows that concentrations are higher on the west side of Mandela Parkway than on the east side. In general, locations to the west of Mandela Parkway are in closer proximity to the I-880 reroute. We do not observe linear decreases in concentrations from east to west of the Mandela Parkway, because of the varying distances separating it from the I-880 reroute, including a freeway segment that intersects with Mandela Parkway at its south end. Overall, this analysis quantitatively substantiates claims that freeway rerouting reduces the air quality burden on the residents of West Oakland.

### 3.3. Neighborhood Measures

#### 3.3.1. Population Density

**Figure 4:**
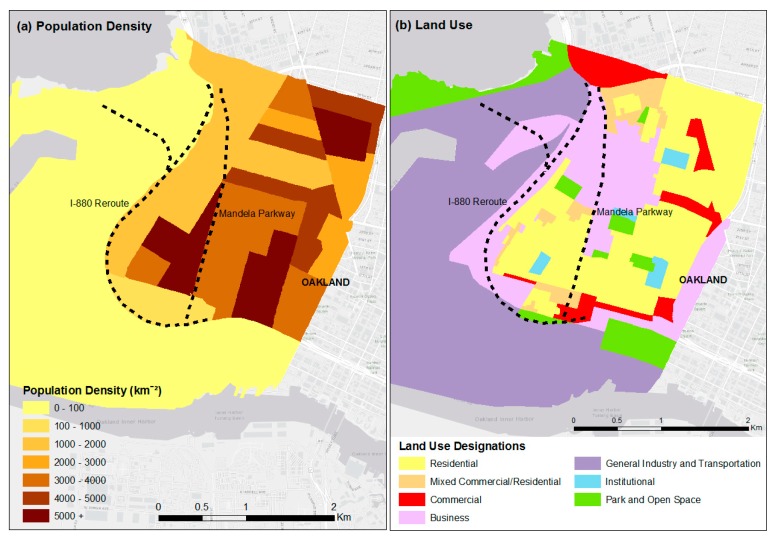
(**a**) Population density (people per km^2^) in West Oakland in 2010. (**b**) Current West Oakland zoning land use designations. Dashed black lines represent the centerlines of the I-880 Reroute (left) and Mandela Parkway (right).

Residents advocated for the replacement of the original Cypress Freeway alignment with a boulevard and relocation of the freeway to more industrial areas of West Oakland, instead of rebuilding along the original alignment, which ran through residential areas. As shown in Figure 4a, the population density in West Oakland is much more concentrated around Mandela Parkway than the I-880 reroute, indicating that, on average, residents experience air quality benefits resulting from freeway rerouting (Figure 2 and Figure 3). Figure 4b shows a map of land use zoning designations for West Oakland, based on data obtained from the City of Oakland [53]. Residential use and industry, commercial, and truck-related uses account for approximately 60% and 23% of the land area in West Oakland, respectively [54]. One primary area of residential use is along the southern portion of Mandela Parkway, which corresponds to the area with the highest population density (Figure 4a), while industrial uses are concentrated along the northern portion of Mandela Parkway. This difference in land use explains the difference in population density between the two sections of Mandela Parkway.

#### 3.3.2. Neighborhood Change

Table 1 presents the averages for the West Oakland demographic, socioeconomic, and housing indicators in 1990 and 2010. In 2010, West Oakland had lower proportions of nonwhite and low-income populations than in 1990. As shown in Table 2, there was a substantial decrease in the Black population in West Oakland (−23%), with a significantly larger decline along the Mandela Parkway (−28%). Reductions in the Black population may be due to increases in the cost of housing. The median home value increased by 136% in West Oakland and 184% in the area along the Mandela Parkway. Table 1 indicates that median home values were lower along the Mandela Parkway than in West Oakland in 1990, but significantly higher in 2010. Larger property value increases along the Mandela Parkway compared to West Oakland as a whole may be due to the freeway rerouting and conversion to the street-level boulevard, which would be consistent with previous studies [2,22,23,24]. In order to support this evidence of environmentally driven neighborhood change, further analysis, using a hedonic pricing model, is needed, to empirically attribute the larger rise in property values to the street-level boulevard. Table 1 and Table 2 also indicate that, during this same period, there was an increase in the Latino population. The percentage change of nonwhite residents can ignore such an inter-ethnic shift [55], which is important to consider when investigating environmental benefits for existing residents. 

Table 2 suggests that gentrification occurred throughout West Oakland, including the area within 250 m of Mandela Parkway, between 1990 and 2010. This is reflected by decreases in the percentage of nonwhite residents, percentage of residents living in poverty, and percentage of renter-occupied units, along with a growth of median household income, median rent, median home value, and percentage of college educated residents. Table 2 also provides further evidence of environmental gentrification along Mandela Parkway. In addition to a significantly sharper decrease in the Black population compared to West Oakland, this area had larger increases in median household income and median home value, and a larger decline in the percentage of residents living in poverty. The racial and socioeconomic changes along the Mandela Parkway occurred without land use changes over the same period. Land use projections in the West Oakland Specific Plan also show continued industrial and mixed-use zoning designations along the northern portion of the Mandela Parkway [54]. A case study of the Greenpoint neighborhood in Brooklyn found that retaining industrial zoning after environmental cleanup and green space creation helps prevent residential development that drives out long-term working-class residents [56]. In the present case, maintaining industrial land uses did not stop market-based processes of neighborhood change.

The area along the Mandela Parkway had smaller increases in median rents (19% versus 30%). One potential factor is the presence of affordable housing. There are three affordable housing sites in the southern portion of Mandela Parkway [54], where the residential population along Mandela Parkway is concentrated (Figure 4a). Preserving affordable housing can reduce the displacement of long-time and low-income residents [57].

## 4. Conclusions

West Oakland residents saw rerouting of the Cypress Freeway and replacement with a street-level boulevard as an opportunity to mitigate the air pollution burden that freeway construction had caused in their neighborhood and increase access to urban green space. Our air pollution maps and distance–decay curves reveal that rerouting the Cypress Freeway resulted in substantial reductions in annual average NO_x_ and BC concentrations in the middle of West Oakland when compared to the Cypress Freeway rebuild-in-place scenario. These air quality benefits highlight the importance of roadway types planned through residential neighborhoods, such as freeways and designated truck routes. We observe that the new freeway route still impacts air pollutant levels in the Mandela Parkway corridor, so it is critical to select an alternative route that does not have segments in close proximity to residential areas. Limitations may be present in port communities and communities impacted by goods-movement activities. 

Environmental justice activism sometimes has unintended paradoxical consequences, where efforts to improve a neighborhood make existing residents vulnerable to displacement [21]. This displacement is facilitated through economic revitalization efforts that do not prioritize the needs of existing residents. While the urban freeway was thought of as a tool for urban revitalization by mid-century transportation planners, the removal and rerouting of the urban freeway are viewed as opportunities for redevelopment. An investigation of West Oakland indicates that freeway rerouting and construction of a street-level boulevard result in some environmental gentrification, with property value increases and the displacement of long-time Black residents, similar to freeway removal and tunneling. To ensure existing residents benefit from the air pollution reductions caused by freeway rerouting, affordable housing and other anti-displacement strategies, such as inclusionary zoning and renter protections, should be instituted [58].

There are some limitations in our analysis methods. Traffic counts on the Mandela Parkway were based on a short-duration traffic survey. These counts were extrapolated to annual counts, using temporal allocation factors from McDonald et al. [37] that were derived from freeway traffic count data. Although traffic activity profiles can vary by roadway type [59], data on arterial traffic patterns were not available. More extensive traffic count data on local arterials is needed to improve estimates of the air quality impacts of freeway rerouting. Additionally, our analysis of neighborhood change was conducted at the tract level. Using an area-weighting method to estimate demographic and socioeconomic variables along the Mandela Parkway introduces error. As freeway removal and replacement with a street-level boulevard receives increased attention as a contemporary urban transportation policy, due to aging freeway infrastructure [10,60], it is critical to accurately determine who benefits from such urban green space projects.

## Figures and Tables

**Table 1:** Comparison of racial and socioeconomic composition for all tracts in West Oakland, and within 250 m of the Mandela Parkway alignment in 1990 and 2010.

	1990		2010	
	West Oakland	250 m of Mandela	West Oakland	250 m of Mandela
% Nonwhite	89.3	92.7	77.8	83.6
% Black	66.4	72.8	43.2	44.5
% Latino	13.1	11.8	15.6	21.4
Median Household Income ^a^	$27,399	$22,869	$33,119	$33,790
% Poverty ^b^	67.2	73.2	53.2	53.7
% Renter Occupied	81.4	76.5	74.1	72.4
Median Gross Rent ^a^	$644	$757	$859	$903
Median Home Value ^a^	$161,753	$154,366	$371,275	$415,425 *
% College Educated ^c^	8.1	8.2	24.8	20.8

^a^ In 2010 inflation-adjusted dollars ^b^ Percentage of households with income less than twice the poverty level ^c^ Includes college and advanced degrees * *p* < 0.10.

**Table 2:** Change in racial and socioeconomic composition between 1990 and 2010 for all tracts in West Oakland, and within 250 m of the Mandela Parkway alignment.

	West Oakland	250 m of Mandela
Change in % Nonwhite	−11.4	−9.1
Change in % Black	−23.2	−28.3 *
Change in % Latino	2.5	9.6
Increase in Median Household Income ^a^	34.6%	54.5%
Change in % Poverty ^b^	−13.9	−19.5
Change in % Renter Occupied	−7.3	−4.1
Increase in Median Gross Rent ^a^	29.7%	19.3%
Increase in Median Home Value ^a^	136%	184%
Change in % College Educated ^c^	16.8	12.6

^a^ In 2010 inflation-adjusted dollars ^b^ Percentage of households with income less than twice the poverty level ^c^ Includes college and advanced degrees * *p* < 0.10.
